# Multi-Functional Materials Based on Cu-Doped TiO_2_ Ceramic Fibers with Enhanced Pseudocapacitive Performances and Their Dielectric Characteristics

**DOI:** 10.3390/polym14214739

**Published:** 2022-11-04

**Authors:** Petronela Pascariu, Mihaela Homocianu, Loredana Vacareanu, Mihai Asandulesa

**Affiliations:** ”Petru Poni” Institute of Macromolecular Chemistry, 41A Grigore Ghica Voda Alley, 700487 Iasi, Romania

**Keywords:** electrospinning, titanium dioxide (TiO_2_) and Cu (0.5, 1, 2%)-doped TiO_2_, electrochemical performance, cycle life, supercapacitors

## Abstract

In this work, pure TiO_2_ and Cu (0.5, 1, 2%)-doped TiO_2_ composites prepared by electrospinning technique followed by calcination at 900 °C, and having high pseudocapacitive and dielectric characteristics were reported. These nanocomposites were characterized by scanning electron microscopy, X-ray diffraction, and dynamic water sorption vapor measurements. The structural characterization of these nanostructures highlighted good crystallinity including only the rutile phase. The electrochemical characteristics were investigated by cyclic voltammetry and galvanostatic charge–discharge measurements, which were performed in a KOH electrolyte solution. Among the Cu-doped TiO_2_ nanostructures that were prepared, the one containing 0.5% Cu exhibited superior electrochemical properties, including high specific gravimetric capacitance of 1183 F·g^−1^, specific capacitance of 664 F·g^−1^, energy density of 45.20 Wh·kg^−1^, high power density of 723.14 W·kg^−1^, and capacitance retention of about 94% after 100 cycles. The dielectric investigation shows good dielectric properties for all materials, where the dielectric constant and the dielectric loss decreased with the frequency increase. Thus, all the interconnected studies proved that these new materials show manifold ability and real applicative potential as pseudocapacitors and high-performance dielectrics. Future work and perspectives are anticipated for characterizing electrochemical and dielectric properties for materials including larger amounts of Cu dopant.

## 1. Introduction

Nowadays, electrochemical capacitors used as energy storage devices are attracting special attention due to their fast charge and discharge characteristics, high power density, low cost, long cycle life, and environmental friendliness [1,2]. One-dimensional (1D) nanostructures have superior properties (high aspect-ratio structure, chemical stability, large specific surface area, excellent electronic or ionic charge transfer [3]) compared to 0-dimensional and bulk materials [4,5], where the former is successfully used in supercapacitors and electrochemical energy storage applications. There are a wide variety of oxide semiconductors, including TiO_2_, MnO_2_, NiO, MoO_3_, Co_3_O_4_, etc. used as electrodes for supercapacitor applications [6,7]. It is known that TiO_2_ is an excellent dielectric material with a faradaic charge storage behavior [8,9], is cost-effective, and is found in abundance, but when TiO_2_ alone is used as a supercapacitor electrode, its behavior is relatively poor [10]. Therefore, combining TiO_2_ with a different metal or nonmetal (Cu [11], Ni [12], Mn [13], Co [14]) can lower the bandgap energy, improve the optical response, and increase the charge carrier concentration, which all enhance the supercapacitive properties and power conversion efficiency of the new system [11,15,16]. For example, Krishnan et al. found that Ni as a dopant in TiO_2_ nanofibers increases the conductivity and improves the specific capacitance from 40 to 179 F·g^−1^, for current density 1 A·g^−1^ [17]. Another group reported improved specific capacitance after TiO_2_ was doped with 2% Ta. They found that the specific capacity value for 2% Ta doped TiO_2_ nanofibers was 199 F·g^−1^ compared to TiO_2_, which was 111 F·g^−1^ for a scan rate of 5 mV/s in 1M H_2_SO_4_ electrolyte. Additionally, these materials have shown excellent cycle stability (about 100% retention in specific capacity after 3000 cycles) [18].

The method of preparation, morphology, and the related structure of the TiO_2_/metals play an important role in the electrochemical profile/performance of these materials [19]. Therefore, 1D materials are attractive for this field due to their advantages as they have a large specific surface area, the transport of electrons occurs in one direction, and the construction of the device is easier [20]. The most important methods for obtaining these materials are electrospinning, electrodeposition [21], and anodization [22]. On the other hand, electrospinning is a relatively easy technique, versatile, and a low-cost method. Composite nanofibers with controlled diameters, adjustable compositions, high porosity, low density, and complex organic/inorganic structures with large surfaces were obtained [23]. Lal et al. fabricated a novel structure of Cu/CuO/PCNF/TiO_2_ composites that are uniformly covered by TiO_2_ nanoparticles and synthesized using the electrospinning method together with a hydrothermal technique, followed by air stabilization and carbonization processes to enhance the performance. These developed electrode materials showed a high specific capacitance value of 330 F·g^−1^ at a current density of 1 A·g^−1^ with 78.8% capacitance retention for up to 10,000 cycles and, simultaneously, a high energy density of 45.83 Wh·kg^−1^ at a power density of 1.27 kW·kg^−1^ [24]. Recently, Kumar et al. synthesized asymmetric Ag–Cu_2_O hybrid nanoparticles on graphene substrates by the hydrothermal method as an efficient electrode material for hybrid supercapacitors. They reported a specific capacitance of approx. 812 F·g^−1^, high energy density of 285 W·kg^−1^, and power density of 27.2 Wh·kg^−1^. The corresponding measurements were carryout at a current density of 0.5 A·g^−1^. Additionally, these systems showed good stability up to 2000 cycles [25].

In an attempt to enlarge the library of metal-oxide semiconductors based on TiO_2_, we recently reported on the design and synthesis of Cu-doped TiO_2_ materials with novel conceptual electrochemical, pseudocapacitive, and electrical conductivity properties that were comparatively studied with regard to the most advantageous combination and synergism of the constitutive structural elements. The findings reported in this previous study prompted further investigations in order to understand how the amount of Cu dopant influences different pseudocapacitance and dielectric parameters. Therefore, pure TiO_2_ and Cu-doped TiO_2_ nanocomposites were prepared by electrospinning technique followed by calcination at 900 °C. The structural confirmation was monitored by scanning electron microscopy (SEM), X-ray diffraction (XRD), and dynamic water sorption vapor (DVS) measurements. To highlight the main features and applicative potential of these new materials, the electrochemical characteristics were investigated by cyclic voltammetry (CV) and galvanostatic charge–discharge (GCD) measurements. Additionally, the dielectric and conductivity properties of TiO_2_ and Cu-TiO_2_ nanostructures were investigated. Thus, these investigations provided the necessary tools to build a pathway for understanding the influence of Cu dopant on the smart behavior of resulting nanocomposites. In the end, all the interconnected studies proved that these new materials show manifold ability and real applicative potential as pseudocapacitors and high-performance dielectrics.

## 2. Materials and Methods

### 2.1. Materials

Nanostructures of pure TiO_2_ and Cu-doped TiO_2_ were obtained by the electrospinning method using identical preparation parameters, but calcined at 900 °C according to our previous work [26]. Titanium (IV) isopropoxide (TTIP) (~97%, CAS: 546–68–9), copper acetate [Cu(CH_3_COO)_2_⋅H_2_O] (≥98%, CAS: 6046–91–1), glacial acetic acid (CH₃COOH) (99–100%, CAS: 64–19–7), ethanol (96%, CAS: 64–17–5), polyvinylpyrrolidone (PVP) (Mw = 1.300.000, CAS: 9003–39–8), polyvinylidene fluoride (PVDF) (Mw = 275.000 g/mol, CAS: 24937–79–9), potassium hydroxide (KOH) (≥85%, CAS: 1310–58–3), N,N-dimethylformamide (DMF) (99.8%, CAS: 68–12–2); all the precursors used in obtaining these materials were purchased from Sigma-Aldrich Co. (Taufkirchen, Germany) and used without further purification. Conductive carbon black (size = 20 nm, CAS: 1333-86-4) was purchased from PowderNano.

### 2.2. TiO_2_ and Cu-Doped TiO_2_ Composite Preparations

**Scheme 1:**
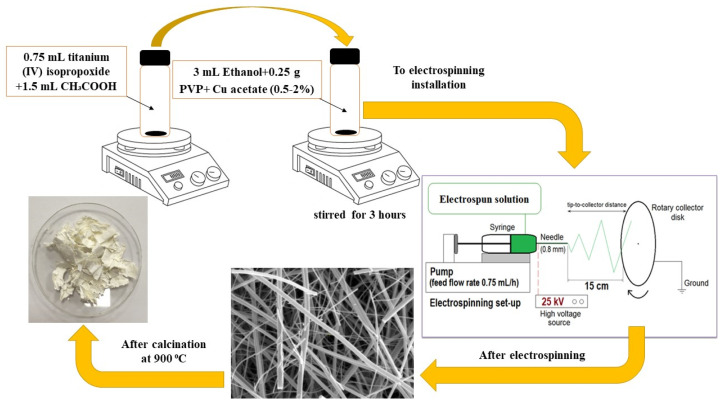
Preparation of the pure and Cu-doped TiO_2_ nanostructures.

The electrospinning method was used to prepare pure TiO_2_ and Cu-doped TiO_2_ nanostructures according to the procedure described in a previous paper, but calcined in air at 900 °C, with a 15 °C/min heating rate [26]. Briefly, pure and doped nanofibers were prepared using electrospun solutions, composed by mixing two solutions, namely: 0.75 mL of TTIP in 1.5 mL acetic acid (solution 1) and 0.25 g PVP in 3 mL ethanol (solution 2). This mixed solution was used to obtain the pure TiO_2_ blank sample (T2). Cu-doped TiO_2_ composites were obtained by adding to solution 2 various amounts (0.5, 1, and 2% by weight) of Cu acetate. These two solutions were mixed and the resulting homogeneous solution was transferred to an electrospinning syringe. All materials were prepared using a homemade electrospinning installation according to our previous studies [27]. Finally, the obtained nanofibers were calcined at 900 °C for 4 h. The samples prepared according to steps from Scheme 1 were tagged as T2 for pure TiO_2_, CT2 for 0.5% Cu-TiO_2_, CT3 for 1% Cu-TiO_2_, and CT4 for 2% Cu-TiO_2_.

### 2.3. Characterization of the Prepared Materials

A Rigaku SmartLab-9kW (Rigaku Corporation, Tokyo, Japan) diffractometer was used to record the XRD patterns of samples and to examine their crystalline structure. The morphological analysis of the Cu-doped TiO_2_ materials was investigated using a Verios G4 UC Scanning Electron Microscope (Thermo Fisher Scientific, Brno, Czech Republic) equipped with an energy dispersive spectrometer (EDS, EDAX Octane Elite). A fully automated gravimetric analyzer, IGAsorp, supplied by Hiden Analytical, Warrington (UK), with an ultrasensitive microbalance, was used to measure the dynamic water vapor sorption capacity of the samples as the weight changes with variation in humidity at a constant temperature. Each sample was dried in flowing nitrogen (250 mL/min) until the weight of the sample was in equilibrium at RH < 1%. Experiments were carried out at 25 °C in the relative humidity (RH) range of 0–90%, with 10% humidity steps, each having a pre-established equilibrium time between 40 and 60 min (minimum time and time out, respectively). The Brunauer–Emmett–Teller (BET) method was used to calculate the specific surface areas of the samples [28]. Broadband dielectric spectroscopy measurements were recorded with a Novocontrol Concept 40 Broadband Dielectric Spectrometer device. The dielectric spectra were recorded isothermally in broad frequency (between 1 Hz to 1 MHz) and temperature (between −150 and 200 °C) ranges. Alternating electrical field oscillations were furnished by an Alpha-A frequency analyzer, and the temperature was controlled to better than 0.1 °C with a Quatro Cryosystem device. The powder samples were obtained as pressed circular pellets with 13 mm diameter and ca. 0.25 mm thick. After preparation, the samples were sandwiched between two cylindrical gold-plated electrodes (furnished by Novocontrol, Montabaur, Germany) and then mounted between the electrodes of the BDS active sample cell.

### 2.4. Electrochemical Characterization 

The electrochemical measurements were performed using a Voltalab PST050 electrochemical workstation with a three-electrode system using a 1 M KOH electrolyte solution at room temperature. A platinum wire was used as the auxiliary electrode, Ag/AgCl as the reference electrode, pure and Cu-doped TiO_2_ on nickel foam as the working electrode. This electrode was prepared according to the following protocol: 80 wt% of TiO_2_ and Cu (0.5, 1, 2%)-doped TiO_2_ nanostructures, 10 wt% of carbon black, and 10% PVDF dissolved in DMF. These mixtures were dropped on Ni foam supports (previously cleaned with ethanol in an ultrasonic bath) and then dried for 12 h at 60 °C [29]. In our experiments, the amounts of pure TiO_2_ and Cu-doped TiO_2_ on the Ni foam electrode were approximately 5.98, 4.84, 6.48, and 6.25 mg, respectively. The specific capacitance of pure and Cu-doped TiO_2_ composites was studied by galvanostatic charge–discharge measurements recorded for a potential between 0 and 0.7 V at a current of 10 mA.

## 3. Results

### 3.1. XRD Analysis

**Figure 1:**
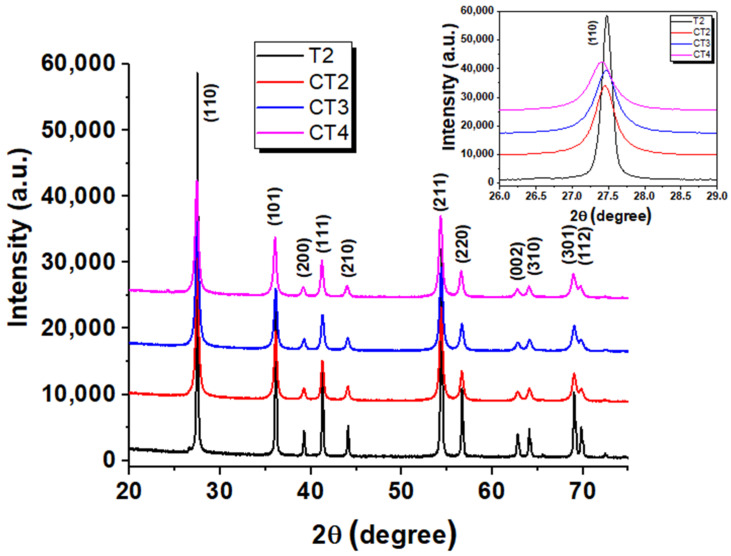
XRD diffraction profiles of pure TiO_2_ (T2) and Cu (0.5, 1, 2%)-doped TiO_2_ (CT2-CT4) composites.

The crystalline structure of the prepared materials was examined by XRD measurements. XRD data were collected in the 2θ range from 20 to 80° with a scanning rate of 1°/min and a step size of 0.01°. Figure 1 shows the diffraction profiles of pure TiO_2_ and Cu (0.5, 1, 2%) doped TiO_2_ calcined at 900 °C. These nanostructures showed good crystallinity containing only the rutile phase. It is known that rutile is the predominant phase during the calcination of TiO_2_ materials at 900 °C [30]. All studied composites highlighted diffraction peaks at 27.47°, 36.03°, 39.23°, 41.28°, 44.09°, 54.35°, 56.66°, 62.79°, 64.07°, 69.07°, and 69.80°, corresponding to the rutile phase of TiO_2_ and associated with the crystallographic planes (110), (101), (200), (111), (210), (211), (220), (002), (310), (301), and (112), respectively. We note that no additional peaks were identified, which means that the Cu ions were fully integrated into the rutile structure of TiO_2_ [14]. Additionally, composites prepared at different Cu concentrations have slight leftward shifts in the peak position compared to pure TiO_2_ (inset of Figure 1). Moreover, we found that the intensity of the diffraction peaks decreases with increasing Cu concentration. The most important structural parameters, including the average crystallite size (D), unit cell parameters (a, c), Ti-O-Ti bond length (L), unit cell volume values (V), and the micro-deformation (ε) were determined using different relationships defined in our previous studies [31,32], while the obtained values are listed in Table 1. The average D values decreased significantly for Cu-doped TiO_2_ from about 40 to 20 nm, due to the inhibition of crystallite growth after the inclusion of copper ions into the crystal structure of TiO_2_ [14,33]. Moreover, the cell parameter increases after Cu doping in the lattice together with an increase in volume, while the cell parameter c decreased slightly, demonstrating that Cu was successfully incorporated into the lattice. The full width at half-maximum (FWHM) values increased from 0.17 to 0.42 with increasing copper content from 0.5 to 2% in the composites [34].

### 3.2. Morphological Characterization

**Figure 2:**
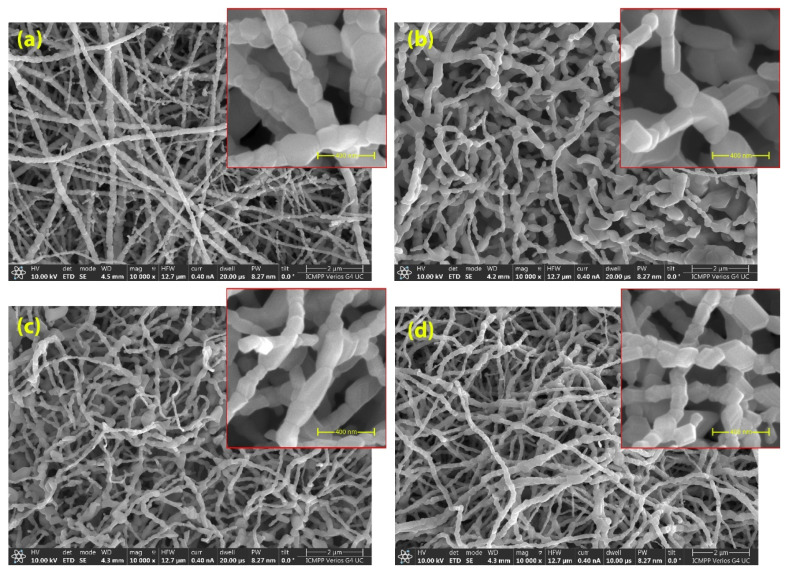
SEM images for pure TiO_2_ (**a**) and 0.5% (**b**), 1% (**c**), and 2% (**d**) Cu-doped TiO_2_ composites.

The surface morphology of pure TiO_2_ and the Cu-doped TiO_2_ nanostructures were examined by SEM imaging, as shown in Figure 2. The nanofibers were round, fully interconnected, and exhibited a uniform diameter distribution (Figure 2a). These images depicted the presence of Cu dopant that was negligibly agglomerated and distributed along the axial direction of the nanofibers (Figure 2b,d). The presence of Cu dopant was evidenced by the EDS spectra presented in Figure S1 in the Supplementary Materials. The presence of platinum in the EDS analysis results from the sample preparation for SEM/EDS measurements. The atomic percentage of Cu recorded in the material varied between 0.31% and 0.57%, depending on the amount of precursor solutions it contained in the initial composition. Moreover, no other impurities were found. As can be seen from the EDS mapping images shown in Figure S2, these materials have good homogeneity of elements. According to the histograms reported in Figure S3, the average size of the fiber diameters varies between 150 and 200 nm. These histograms were obtained by measuring 100 fiber diameters for each material and prepared using Image-J software (Java 1.8.0_345 (64-bit)).

### 3.3. Sorption and Desorption Isotherms

**Figure 3:**
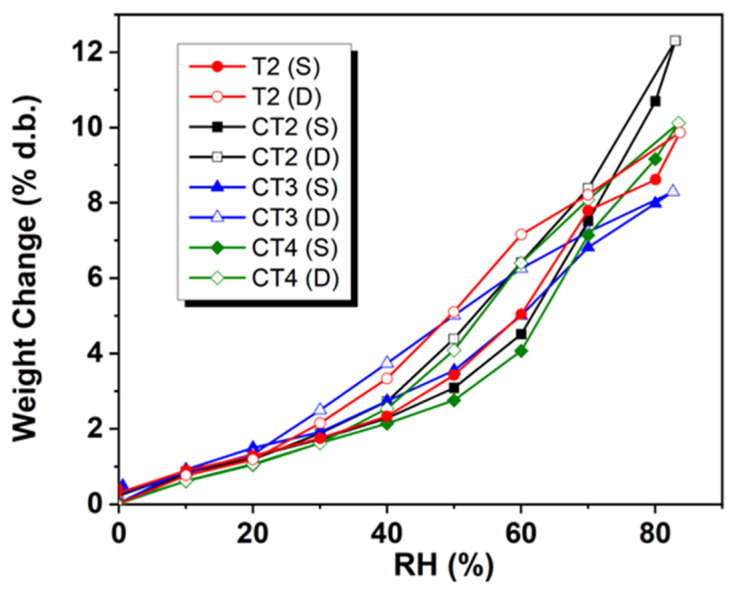
Adsorption (S) and desorption (D) isotherms for pure TiO_2_ and Cu-doped TiO_2_ composites.

It is known that materials with a large specific surface area can provide several active surface places and facilitate the transport of ions, improving the electrochemical performance of the materials [14]. The adsorption and desorption isotherms of pure TiO_2_ and Cu-doped TiO_2_ nanostructures are shown in Figure 3. From these isotherms, it can be seen that water desorption is faster than water sorption for all samples, which highlights their hysteresis behavior. The water adsorption–desorption isotherms (Figure 3) show the dependence between the equilibrium water content of the composites and relative humidity (RH), and are used to estimate the sorption capacity, average pore size, and specific surface area of the prepared materials (Table 2). Below 30% RH, the mass changes were negligible compared to the initial weight and showed no significant modifications when the Cu dopant content was varied (Figure 3). At more than 30% RH, the mass changes increased exponentially with RH values. At 80% RH, the mass change was higher for the 0.5% Cu-TiO_2_ composite. The sorption capacities of the prepared composites were correlated with their hysteresis behavior and were found to have the highest values for the 0.5% Cu-TiO_2_ composite (12.30% d.b.). From Table 2, it can be seen that the addition of copper dopant increased the pore size (from 1.7 nm for pure TiO_2_ to 4.31 nm for 0.5% Cu-TiO_2_) and their sorption capacity values. The BET surface area (Table 2) for pure TiO_2_ is 112 m^2^/g, but with increasing Cu-doping concentration, a decrease in surface area values was observed due to a larger ionic radius of Cu ions (0.72 Å) than that of Ti ions (0.68 Å). Ganesh et al. also reported that upon increasing the Cu-doping concentration in TiO_2_ thin film, BET surface area values decreased [35]. The lowest surface area value (57.2 m^2^/g,) is for 0.5% Cu-TiO_2_ having the largest average pore size (4.31 nm, Table 2).

### 3.4. Electrochemical Characterization

**Figure 4:**
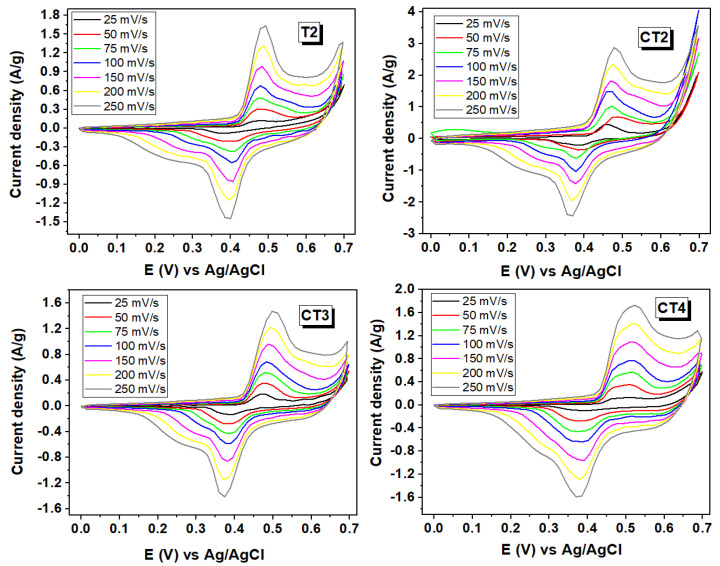
Cyclic voltammetry curves of pure TiO_2_ (**T2**) and 0.5% Cu (**CT2**), 1% Cu (**CT3**), 2% Cu (**CT4**) doped TiO_2_ nanostructures, at different scan rates.

The electrochemical performance of Cu-doped TiO_2_ nanostructures compared to pure TiO_2_ was evaluated by CV, GCD, and stability measurements. Figure 4 shows the CV curves, which demonstrate the pseudocapacitive behavior given by the redox reaction pairs (with good redox symmetry) [36] and good stability recorded for all samples. According to these curves, the capacitance characteristics are dominated by faradaic reactions and are different from those of double-layer electric capacitors [37]. The area of the CV curves increases significantly from pure TiO_2_ electrodes to Cu-doped TiO_2_ (Figure 4), suggesting better capacitive behavior of the composite materials compared to pure TiO_2_. Additionally, as the scan rate increased, a similar shape and an increase in anodic peak currents (during direct and reversed sweep) were observed (Figure 4), demonstrating reversibility [38] and good capacitive behavior. The pseudocapacitance behavior may be due to the interconversion between oxidation states of Cu(I) and Cu(II) [25] representing anodic and cathodic peaks (Figure 4). From the CV curves, it can be observed that the magnitude (current densities) of the oxidation (0.37 V) and reduction (0.47 V) peaks are higher for 0.5% Cu-TiO_2_ compared to other samples (CT3), indicating higher capacitance for this sample.

**Figure 5:**
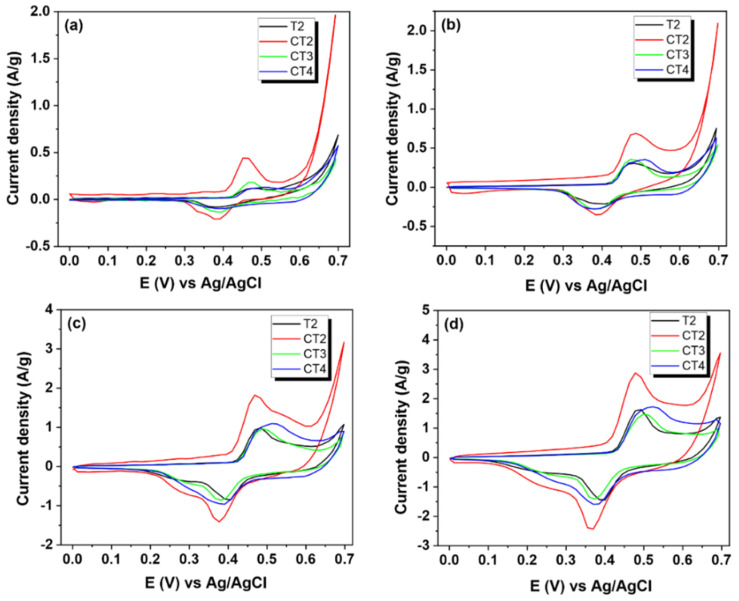
Cyclic voltammetry curves for TiO_2_ (T2) and Cu (0.5%, 1%, 2%)-doped TiO_2_ (CT2-CT4) recorded at 25 mV/s (**a**); 50 mV/s (**b**); 150 mV/s (**c**); and 250 mV/s (**d**) scan rates.

Figure 5 shows the cyclic voltammetry comparison curves of pure TiO_2_ and Cu (0.5, 1, 2%)-doped TiO_2_ composites recorded at scanning rates of 25, 50, 150, and 250 mV/s. All these curves exhibited oxidation and reduction peaks positioned at the potentials of 0.46 and 0.52 V, whose intensities increased with increasing scanning speed values. The area under the CV curves for the 2% Cu-TiO_2_ composite at 250 mV/s was larger than the area under the CV curves for TiO_2_, 0.5% Cu-TiO_2_, and 1% Cu-TiO_2_ (Figure 5), indicating an enhanced capacitance for this composite.

**Figure 6:**
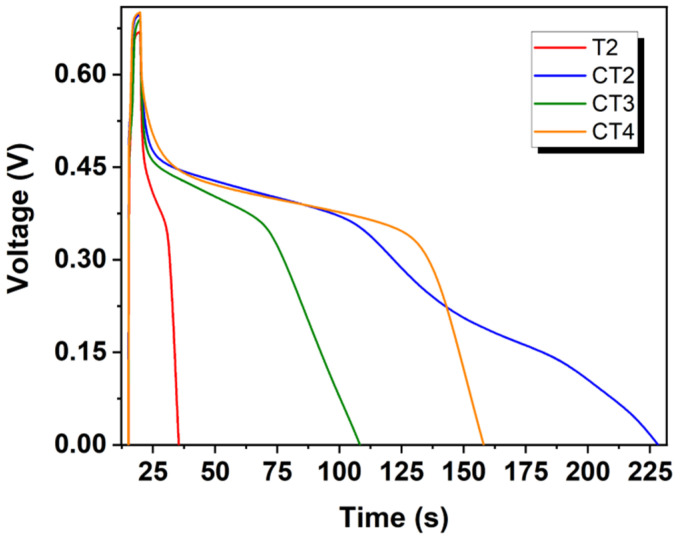
Galvanostatic charge–discharge curves of pure TiO_2_ (T2) and Cu-TiO_2_ (CT2-CT4) composites.

To study in detail the electrochemical properties of pure TiO_2_ and Cu-doped TiO_2_ composites, we employed the investigation of the GCD curves. Figure 6 shows the GCD curves of pure TiO_2_ and Cu-TiO_2_ composites that display asymmetry, suggesting that the materials undergo redox reactions according to cyclic voltammetry curves (Figure 5). After this plateau, it can be seen that the voltage decreases sharply due to the internal resistance of the material in the part of the discharge curve [39]. Moreover, the GCD curves of the composites exhibited asymmetry and good reversibility, indicating faradaic pseudocapacitance behavior [40].

Based on the galvanostatic charge–discharge curves, the specific capacitance (CSP, F·g^−1^), [18] the energy density (*E*, Wh·kg^−1^) [41], and power density (*P*, W·kg^−1^) [42] were calculated by applying the following relationships:

(1) $$C_{SP}=\frac{\int IV}{2mV}\;(\mathrm{gravimetric})\;$$

(2) $$C_{SP}=\frac{I\times t}{V\times m}$$

(3) $$E=\frac{C_{SP}\times \Delta V^{2}}{7.2}$$

(4) $$P=\frac{E\times 3600}{\Delta t}$$

where *C_SP_*, *I*, Δ*t*, Δ*V*, and m are the specific capacitance, the charge–discharge current (A), the discharging time (s), the applied potential window (*V*), and the active mass of the electrode (g), respectively.

**Figure 7:**
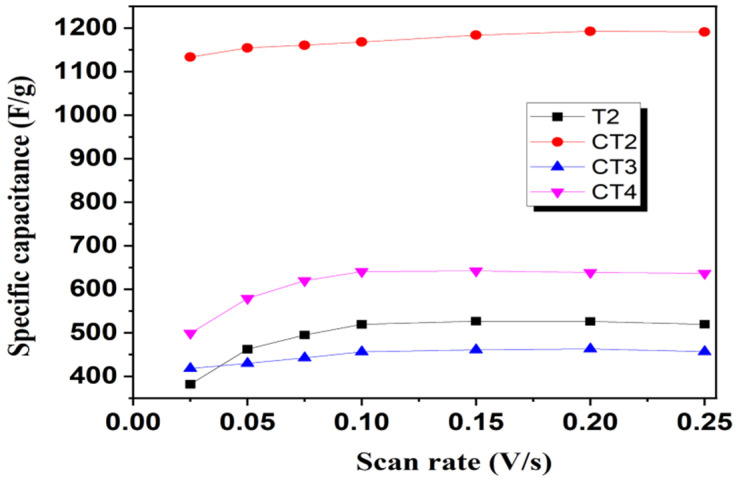
The evolution of specific gravimetric capacitance at the different scan rates for pure TiO_2_ and Cu-doped TiO_2_ composites.

From galvanostatic charge–discharge measurements, it was found that the 0.5% Cu-doped TiO_2_ (CT2) nanostructure showed a longer charge–discharge time than the other doped materials (CT3, CT4), demonstrating the highest specific capacitance. Several factors can affect the specific capacitance of the electrode materials, including specific surface area, shape, nanostructure size, and conductivity [14]. Figure 7 shows the dependence of specific gravimetric capacitance on scan rates. According to Figure 7 and Table 3, we found that the maximum capacitance value of 1183.89 F·g^−1^ (at 0.15 V/s) corresponds to sample CT2 (with 0.5% Cu). Pure TiO_2_ had a specific capacitance value of 526 F·g^−1^ (at 0.15 V/s), which is higher than previously reported for some other TiO_2_-based electrodes [29,43,44]. For example, Pant et al. [29] reported that TiO_2_ carbon nanofibers can be successfully used as electrodes for supercapacitors due to their outstanding specific properties, including specific capacity (106.57 F·g^−1^) and 84% capacity retention after 2000 cycles. Likewise, He et al. prepared electrode material based on TiO_2_ nanofiber using an electrospinning technique and then treated them in KOH. The specific capacity found for this material improved dramatically from 0.04 F·g^−1^ (for untreated TiO_2_) to 65.84 F·g^−1^ at 1 mV/s [43].

The specific capacitance, energy density, and power density values for all evaluated materials using Equations (1)–(4) are listed in Table 3. From these results, it is clear that Cu-doped TiO_2_ shows better electrochemical performance compared to the pure TiO_2_ nanoparticles, suggesting that copper contributes to the improvement of the electronic conductivity of TiO_2_. Therefore, the highest values for energy density (45.20 Wh·kg^−1^) and power density (723.14 W·kg^−1^) were found for 0.5% Cu-doped TiO_2_ composite.

**Figure 8:**
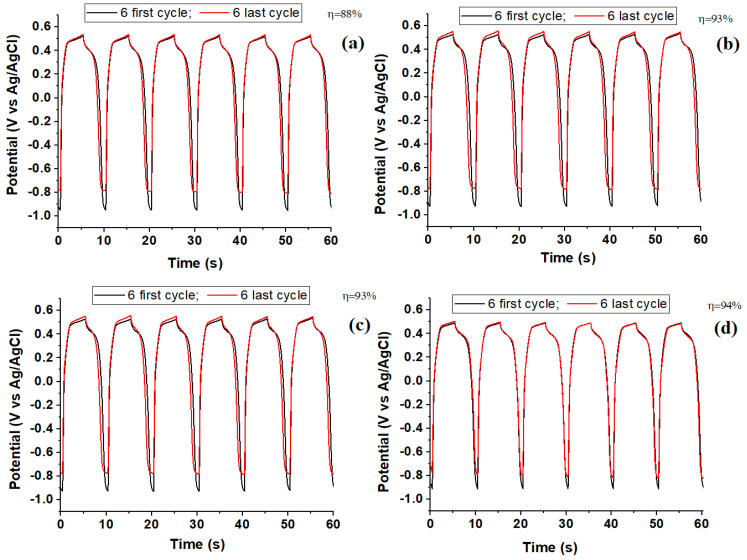
First six cycles and last five cycles of charge–discharge curves for (**a**) TiO_2_, (**b**) 0.5% Cu-TiO_2_, (**c**) 1% Cu-TiO_2_, and (**d**) 2% Cu-TiO_2_ composites.

The life cycle stability is a key characteristic of supercapacitors, so for the pure TiO_2_ and Cu-TiO_2_ electrode materials, this parameter was evaluated by measuring the consecutive charge–discharge curves (Figure 8) for 100 cycles. From this figure, it is clear that the coulombic efficiency of Cu-doped materials (~94%) increased compared to that of pure TiO_2_ (88%), suggesting good stability at 100 cycles. Moreover, the results obtained in this work indicated that the electrochemical performance of Cu-doped TiO_2_ is superior to pure TiO_2_ and is comparable to other related metal-doped TiO_2_ systems found in the literature (Table 4). 

### 3.5. Dielectric Spectroscopy Measurements

**Figure 9:**
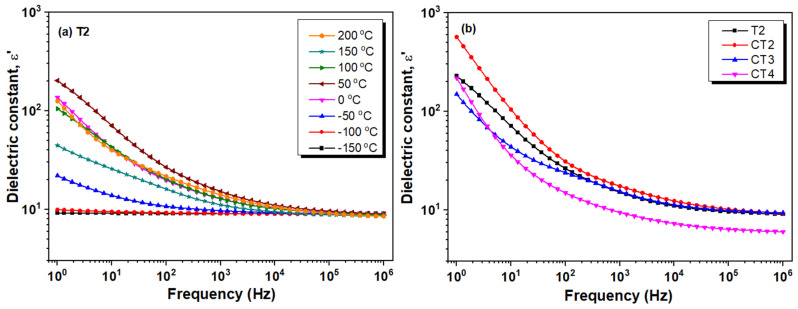
The evolution of dielectric constant as a function of frequency: (**a**) exemplarily for T2 at various temperatures and (**b**) comparatively for all examined samples at room temperature.

To obtain better insights regarding the full potential of these materials in supercapacitors, dielectric spectroscopy measurements were employed. Therefore, Figure 9a shows the behavior of the dielectric constant with the applied field frequency for the T2 sample toward the increasing temperature from −150 to 200 °C. At low temperatures, ε′ is of low magnitude in the entire frequency range (e.g., at −100 °C and 1 kHz, ε′ = 9.1), suggesting a reduced dipolar activity. As temperature increases, the magnitude of ε′ is enhanced, due to the accumulation of thermal energy by the polarizable units. Moreover, at temperatures higher than 0 °C, ε′ shows a pronounced decrease, especially at low frequencies. According to the literature, this behavior of ε′ is produced by the accumulation of charge carriers at the interface between the sample surfaces and the electrodes used to perform the dielectric measurements [46,47]. Undoubtedly, the electrode polarization process of the blocked charges affects the dipolar-type signal. With further increase in frequency, however, the effect of space charge polarization is diminished, revealing a moderate decrease in ε′. Therefore, two different processes are distinguished: (i) the electrode polarization effect manifested at low frequencies and (ii) the dipolar activity of the sample at high frequencies. Following the ε′(f) dependences of all samples (Figure 9b), no clear effect of the copper doping on the dielectric constant was observed.

**Figure 10:**
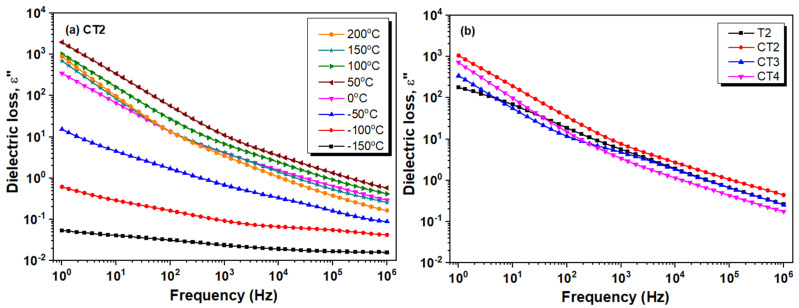
The evolution of dielectric loss as a function of frequency: (**a**) exemplarily for CT2 at various temperatures and (**b**) comparatively for all examined samples at room temperature.

The isothermal plots of dielectric loss ε″ as a function of frequency at different temperatures are retrieved in Figure 10a for the CT2 sample. The magnitude of ε″ is regularly enhanced with increasing temperature in the entire frequency range, suggesting that the polarizable units are closely connected to the temperature changes. On the other hand, ε″ decreases gradually toward increasing frequency and presents a pronounced decrease in magnitude, in particular at low frequencies and high temperatures, in a similar way to the frequency behavior of the dielectric constant parameter. The linear decrease in ε″ at low frequencies suggests the existence of free charges in the material lattice [48,49]. As observed in Figure 10b, ε″ of copper-doped TiO_2_ samples follow similar tendencies, with a linear decrease at low frequencies and a monotonous decrease at higher frequencies. For the T2 sample, the linear decrease at low frequencies is missing, suggesting the absence of the free charge carriers.

**Figure 11:**
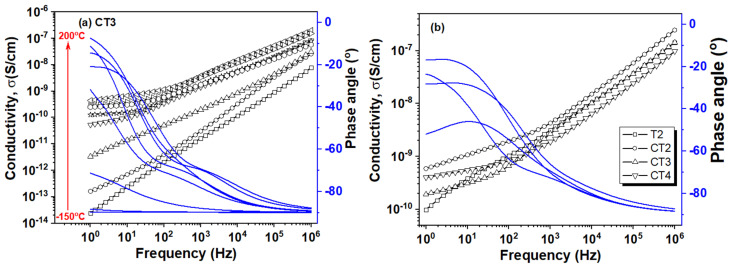
The evolution of conductivity as a function of frequency: (**a**) exemplarily for CT3 at various temperatures and (**b**) comparatively for all examined samples at room temperature.

The behavior of the measured conductivity (σ = 2πε_0_ƒε″, where ε_0_ is the vacuum permittivity and ƒ is the frequency of the applied field) and the phase angle (θ = tan^−1^ (Z_im_/Z_re_), where Z_im_ and Z_re_ are the imaginary and the real components of the impedance) with the frequency at various temperatures are represented in a common diagram in Figure 11a for CT3 sample. At low temperatures, σ regularly increases toward increasing frequency and the corresponding values of θ are detected between 0 < θ(ƒ) ≤ −90°. This capacitive behavior is generally attributed to the dipolar-type relaxation processes of the material. On the other hand, at low frequencies and high temperatures, a change in the slope of the measured conductivity, correlated with the enhancement of the phase angle values close to 0° is observed. This behavior is of resistive type and may be assigned to the presence of mobile charge carriers in the material backbone [50]. The comparative σ(ƒ), as well as θ(ƒ) profiles of all examined samples retrieved in Figure 11b, show that the resistive behavior of measured conductivity is detected only for the prepared copper-doped TiO_2_ samples. This may suggest that copper doping acts as a source of free charge carriers.

**Figure 12:**
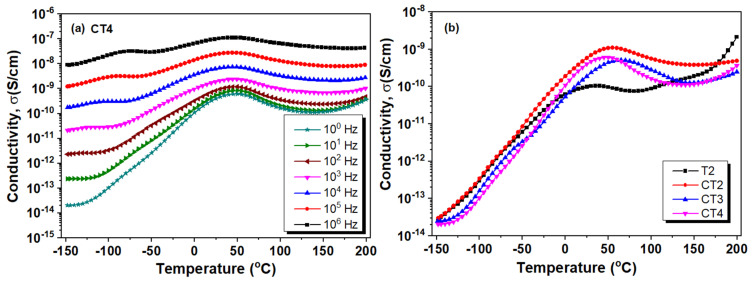
The evolution of conductivity as a function of temperature: (**a**) exemplarily for CT4 at various frequencies and (**b**) comparatively for all examined samples at room temperature.

In Figure 12a, the behavior of conductivity as a function of temperature for various frequencies is represented for the CT4 sample, as a representative example. The magnitude of σ increases gradually with the frequency increase. The conductivity values were further selected at a frequency of 1 Hz and were plotted as a function of temperature in Figure 12b for neat T2 and copper-doped TiO_2_ samples. All examined samples reveal similar temperature behavior. At low temperatures, between −150 °C and around 0 °C, all examined samples reveal a linear increase in conductivity with a similar slope and comparable values of σ. At temperatures higher than 0 °C, an inflection point is detected. It is worth mentioning that the maximum of inflection point is remarkably shifted in copper-doped TiO_2_ samples to higher temperatures, while the maximum inflection point for T2 sample is detected around 35 °C, 55 °C for CT2, 65 °C for CT3, and 48 °C for CT4. Moreover, in the temperature region of the inflection point, the numerical values of conductivity are higher for copper-doped TiO_2_ samples than for neat T2 samples (see Table 5 with σ values retrieved at room temperature).

## 4. Conclusions

The pure TiO_2_ and Cu-TiO_2_ composites were prepared by electrospinning followed by carbonization at 900 °C. The obtained materials showed good crystallinity with the presence of only the rutile phase. Furthermore, the average crystallite size decreased significantly for Cu-doped TiO_2_ from about 40 to 20 nm. The (0.5, 1, 2%) Cu-doped TiO_2_ composites exhibited a higher specific capacitance, better rate capability, and cycling stability compared to pure TiO_2_, although the particle size and pore size were larger and surface area was lower. It was found that the 0.5% Cu-doped TiO_2_ (CT2) nanostructure showed a longer charge–discharge time (227 s) than other doped materials, demonstrating the highest specific capacitance. It was found that 0.5% Cu-TiO_2_ is the optimal dopant concentration in terms of electrochemical and capacitive performances. The maximum capacitance value obtained for this material was 1183.89 F·g^−1^ (at 0.15 V/s). After 100 charge–discharge cycles, the capacitance retention of about 94% for Cu-doped TiO_2_ shows good cycling stability. The dielectric constant, the dielectric loss, and the dc conductivity of materials in the frequency range 10 Hz–1 MHz were measured at different temperatures. With increasing frequency and temperature, the electric conductivity increased. Therefore, regarding its use in pseudocapacitor applications, electrospun Cu-doped TiO_2_ nanostructures exhibit superior electrochemical properties compared to pure TiO_2_. To determine to what extent and how this can influence the electrochemical and dielectric properties, future work and perspectives will be directed toward investigating these characteristics when using larger amounts of Cu dopant.

## Figures and Tables

**Table 1:** The values of the lattice parameters (crystallographic planes (*d_hkl_*), the average crystallite size (D, nm), unit cell parameters (*a*, *c*, Å), Ti-O-Ti bond length (L, nm), and the micro-deformation (ε, %)) and volume (V, Å^3^) of pure TiO_2_ and Cu-doped TiO_2_ nanostructures.

Samples	*2θ* (º)	*(hkl)*	*d_hkl_* (Å)	FWHM (º)	D (nm)	*a* (Å)	*c* (Å)	ε (%)	L (nm)	V (Å^3^)
**T2**	27.47	(110)	3.244	0.17	48.05	4.588	*-*	0.174	4.035	53.90
	36.11	(101)	2.485	0.21	39.67	*-*	2.957	0.203	-	-
**CT2**	27.45	(110)	3.247	0.34	23.98	4.592	-	0.348	4.041	53.99
	36.10	(101)	2.486	0.35	23.80	-	2.957	0.268	-	-
**CT3**	27.47	(110)	3.244	0.42	19.41	4.589	-	0.429	4.037	53.91
	36.11	(101)	2.485	0.38	21.92	-	2.956	0.291	-	-
**CT4**	27.41	(110)	3.251	0.42	19.41	4.598	-	0.431	4.050	54.25
	36.03	(101)	2.491	0.40	20.82	-	2.963	0.308	-	-

**Table 2:** Dynamic water sorption vapor data for the tested materials.

Sample	Sorption Capacity, % d.b.	Average Pore Size, nm	BET Data	
			Area, m^2^/g	Monolayer, g/g
**T2**	9.86	1.77	112	0.031
**CT2**	12.30	4.31	57.2	0.0163
**CT3**	8.30	2.41	69	0.0196
**CT4**	10.10	3.19	63.5	0.0181

**Table 3:** Data obtained from cyclic voltammetry and galvanostatic charge–discharge curves for pure TiO_2_ and Cu-TiO_2_ electrodes.

Electroactive Materials	Specific Gravimetric Capacitance (F·g^−1^)	Specific Capacitance (F·g^−1^)	Energy Density (Wh·kg^−1^)	Power Density (W·kg^−1^)
**T2**	526	84	5.69	585.28
**CT2**	1183.89	664	45.20	723.14
**CT3**	456	235	16.05	540.12
**CT4**	640	361	24.56	559.55

**Table 4:** A comparison of the electrochemical properties of Cu-doped TiO_2_ composites with the properties of other metal-doped TiO_2_ systems reported in the literature.

Material	Preparation Method	Electrolyte	C_SP_ (F·g^−1^)	Specific Energy (Wh·Kg^−1^)	Specific Power (W·Kg^−1^)	CS Retention (%)/Cycle Number	Ref.
Nb-doped TiO_2_		3 M KOH	52	770	16.3	100/5000	[45]
Ta-doped TiO_2_	electrospinning	1M H_2_SO_4_	199 at 5 mV/s	11.25	100.49	100/5000 at a current density of 1 A g^−1^	[18]
Co-doped TiO_2_	solvothermal method	3 M KOH	352 at a current density of 0.5 A/g	-	-	97/3000 at a current density of 0.5 A g^−1^	[14]
Ni-doped TiO_2_	electrospinning	3 M KOH	200 at 2 mV/s	-	-	100/5000	[17]
0.5%Cu-doped TiO_2_	electrospinning	1 M KOH	1183.89 at 0.15 V/s	45.20	723.14	94/100	this work

**Table 5:** Numerical values of dielectric constant, dielectric loss and conductivity were retrieved at room temperature for all examined samples.

Sample	ε′ (1 kHz)	ε″ (1 kHz)	σ (S/cm) (1 Hz)
**T2**	15.1	5.7	1 × 10^−10^
**CT2**	17.5	7.7	6 × 10^−10^
**CT3**	15.4	4.8	2 × 10^−10^
**CT4**	9.4	3.4	4 × 10^−10^

## Data Availability

Not applicable.

## Supplementary Materials

The following supporting information can be downloaded at: https://www.mdpi.com/article/10.3390/polym14214739/s1, Figure S1. EDS spectra of 0.5% Cu (CT2), 1% Cu (CT3), 2% Cu (CT4) doped TiO_2_ composites. Figure S2. Elemental mapping images of 2% Cu- doped TiO_2_ (CT4) composite. Figure S3. Histogram plots of average size diameter distribution for pure TiO2 and 0.5% Cu (CT2), 1% Cu (CT3), 2% Cu (CT4) doped TiO_2_ composites.

## References

[1] Ahirrao D.J., Wilson H.M., Jha N. (2019). TiO_2_-nanoflowers as flexible electrode for high performance supercapacitor. Appl. Surf. Sci..

[2] Da Silva E.P., Rubira A.F., Ferreira O.P., Silva R., Muniz E.C. (2019). In situ growth of manganese oxide nanosheets over titanium dioxide nanofibers and their performance as active material for supercapacitor. J. Colloid Interface Sci..

[3] Tian J., Zhao Z., Kumar A., Boughton R.I., Liu H. (2014). Recent progress in design, synthesis, and applications of one-dimensional TiO_2_ nanostructured surface heterostructures: A review. Chem. Soc. Rev..

[4] Abd-Elnaiem A.M., Hamdalla T.A., Seleim S.M., Hanafy T.A., Aljohani M., Rashad M. (2021). Correction to: Influence of incorporation of gallium oxide nanoparticles on the structural and optical properties of polyvinyl alcohol polymer. J. Inorg. Org. Polym. Mater..

[5] Ali N.A., Abd-Elnaiem A.M., Hussein S.I., Khalil A.S., Alamri H.R., Assaedi H.S. (2021). Thermal and Mechanical Properties of Epoxy Resin Functionalized Copper and Graphene Hybrids Using In-situ Polymerization Method. Curr. Nanosci..

[6] Khedekar V.V., Zaeem S.M., Das S. (2018). Graphene-metal oxide nanocomposites for supercapacitors: A perspective review. Adv. Mater. Lett..

[7] Afif A., Rahman S.M.H., Tasfiah Azad A., Zaini J., Islan M.A., Azad A.K. (2019). Advanced materials and technologies for hybrid supercapacitors for energy storage—A review. J. Energy Storage.

[8] Zhang Z., Xiao F., Guo Y., Wang S., Liu Y. (2013). One-pot self-assembled three-dimensional TiO_2_-graphene hydrogel with improved adsorption capacities and photocatalytic and electrochemical activities. ACS Appl. Mater. Interfaces.

[9] Heng I., Lai C.W., Juan J.C., Numan A., Iqbal J., Teo E.Y.L. (2019). Low-temperature synthesis of TiO_2_ nanocrystals for high performance electrochemical supercapacitors. Ceram. Int..

[10] Guan C., Xia X., Meng N., Zeng Z., Cao X., Soci C., Zhang H., Fan H.J. (2012). Hollow core–shell nanostructure supercapacitor electrodes: Gap matters. Energy Environ. Sci..

[11] Dhonde M., Sahu K., Murty V.V.S., Nemala S.S., Bhargava P., Mallick S. (2018). Enhanced photovoltaic performance of a dye sensitized solar cell with Cu/N Co-doped TiO_2_ nanoparticles. J. Mater. Sci. Mater. Electron..

[12] Wang H., Tang Z., Sun L., He Y., Wu Y., Li Z. (2009). Capacitance performance enhancement of TiO_2_ doped with Ni and graphite. Rare Met..

[13] Ning X., Wang X., Yu X., Zhao J., Wang M., Li H., Yang Y. (2016). Outstanding supercapacitive properties of Mn-doped TiO_2_ micro/nanostructure porous film prepared by anodization method. Sci. Rep..

[14] Qian Y., Du J., Kang D.J. (2019). Enhanced electrochemical performance of porous Co-doped TiO_2_ nanomaterials prepared by a solvothermal method. Microporous Mesoporous Mater..

[15] Soares G.B., Ribeiro R.A.P., de Lazaro S.R., Ribeiro C. (2016). Photoelectrochemical and theoretical investigation of the photocatalytic activity of TiO_2_:N. RSC Adv..

[16] Gupta A., Sahu K., Dhonde M., Murty V.V.S. (2020). Novel synergistic combination of Cu/S co-doped TiO_2_ nanoparticles incorporated as photoanode in dye sensitized solar cell. Solar Energy.

[17] Krishnan S.G., Archana P.S., Vidyadharan B., Misnon I.I., Vijayan B.L., Nair V.M., Gupta A., Jose R. (2016). Modification of capacitive charge storage of TiO_2_ with nickel doping. J. Alloys Compd..

[18] Tyagi A., Singh N., Sharma Y., Gupta R.K. (2019). Improved supercapacitive performance in electrospun TiO_2_ nanofibers through Ta-doping for electrochemical capacitor applications. Catal. Today.

[19] Liang S., Wang X., Qi R., Cheng Y.-J., Xia Y., Müller-Buschbaum P., Hu X. (2022). Bronze-phase TiO_2_ as anode materials in lithium and sodium-ion batteries. Adv. Func. Mater..

[20] Li X., Chen Y., Zhou L., Maia Y.W., Huang H. (2014). Exceptional electrochemical performance of porous TiO_2_–carbon nanofibers for lithium ion battery anodes. J. Mater. Chem. A.

[21] Meier L.A., Alvarez A.E., García S.G., del Barrio M.C. (2015). Formation of Cu and Ni nanowires by electrodeposition. Procedia Mater. Sci..

[22] Mohamed M., Moustafa S., Taha S.A., Abd-Elnaiem A.M. (2018). Morphological characterization and refractive index calculation of anodized titanium (99.7%) foil in HF-ethanol electrolyte. Mater. Res. Express.

[23] Pascariu P., Cojocaru C., Samoila P., Olaru N., Bele A., Airinei A. (2021). Novel electrospun membranes based on PVDF fibers embedding lanthanide doped ZnO for adsorption and photocatalytic degradation of dye organic pollutants. Mater. Res. Bull..

[24] Lal M.S., Lavanya T., Ramaprabhu S. (2019). An efficient electrode material for high performance solid-state hybrid supercapacitors based on a Cu/CuO/porous carbon nanofiber/TiO_2_ hybrid composite. Beilstein J. Nanotechnol..

[25] Kumar M., Chauhan H., Satpati B., Deka S. (2018). Yolk type asymmetric Ag–Cu_2_O hybrid nanoparticles on graphene substrate as efficient electrode material for hybrid supercapacitors. J. Phys. Chem..

[26] Pascariu P., Cojocaru C., Samoila P., Airinei A., Olaru N., Rotaru A., Romanitan C., Tudoran L.B., Suchea M. (2022). Cu/TiO_2_ composite nanofibers with improved photocatalytic performance under UV and UV-visible light irradiation. Surf. Interfaces.

[27] Cojocaru C., Dorneanu P.P., Airinei A., Olaru N., Samoila P., Rotaru A. (2017). Design and evaluation of electrospun polysulfone fibers and polysulfone/NiFe_2_O_4_ nanostructured composite as sorbents for oil spill cleanup. J. Taiwan Inst. Chem. Eng..

[28] Brunauer S., Deming L.S., Deming W.E., Teller E. (1940). On a theory of the van der Waals adsorption of gases. J. Am. Chem. Soc..

[29] Pant B., Park M., Park S.J. (2019). TiO_2_ NPs assembled into a carbon nanofiber composite electrode by a one-step electrospinning process for supercapacitor applications. Polymers.

[30] Rahimi N., Pax R.A., Gray E., Mac A. (2016). Review of functional titanium oxides. I: TiO_2_ and its modifications. Prog. Solid. State Chem..

[31] Pascariu P., Homocianu M., Cojocaru C., Samoila P., Airinei A., Suchea M. (2019). Preparation of La doped ZnO ceramic nanostructures by electrospinning–calcination method: Effect of La^3+^ doping on optical and photocatalytic properties. Appl. Surf. Sci..

[32] Türkyılmaz Ş.Ş., Güy N., Özacar M. (2017). Photocatalytic efficiencies of Ni, Mn, Fe and Ag doped ZnO nanostructures synthesized by hydrothermal method: The synergistic/antagonistic effect between ZnO and metals. J. Photochem. Photobiol. A Chem..

[33] Pascariu P., Cojocaru C., Homocianu M., Samoila P., Dascalu A., Suchea M. (2022). New La^3+^ doped TiO_2_ nanofibers for photocatalytic degradation of organic pollutants: Effects of thermal treatment and doping loadings. Ceram. Int..

[34] Mahmood P.H., Amiri O., Ahmed S.S., Hama J.R. (2019). Simple microwave synthesis of TiO_2_/NiS_2_ nanocomposite and TiO_2_/NiS_2_/Cu nanocomposite as an efficient visible driven photocatalyst. Ceram. Int..

[35] Ganesh I., Kumar P.P., Annapoorna I., Sumliner J.M., Ramakrishna M., Hebalkar N.Y., Padmanabham G., Sundararajan G. (2014). Preparation and characterization of Cu-doped TiO_2_ materials for electrochemical, photoelectrochemical, and photocatalytic applications. Appl. Surf. Sci..

[36] Cheng J.P., Fang J.H., Li M., Zhang W.F., Liu F., Zhang X.B. (2013). Enhanced electrochemical performance of CoAl-layered double hydroxide nanosheet arrays coated by platinum films. Electrochim. Acta.

[37] Poudel M.B., Yu C., Kim H.J. (2020). Synthesis of conducting bifunctional polyaniline@Mn-TiO_2_ nanocomposites for supercapacitor electrode and visible light driven photocatalysis. Catalysts.

[38] Pan C., Sun H., Gao J., Hu Y., Wang J. (2018). Simplified synthesis of 3D flexible reduced graphene oxide-wrapped NiCo_2_O_4_ nanowires for high-performance supercapacitor. Nanosci. Nanotechnol. Lett..

[39] Cao X., Shi Y., Shi W., Lu G., Huang X., Yan Q., Zhang Q., Zhang H. (2011). Preparation of novel 3D graphene networks for supercapacitor applications. Small.

[40] Li P., Ruan C., Xu J., Xie Y. (2019). A high-performance asymmetric supercapacitor electrode based on a three-dimensional ZnMoO_4_/CoO nanohybrid on nickel foam. Nanoscale.

[41] Wang Q., Wang J., Wang H., Zhan J., Zhu Y., Zhang Q., Shen Q., Yang H. (2019). TiO_2_-C nanowire arrays@polyaniline core-shell nanostructures on carbon cloth for high performance supercapacitors. Appl. Surf. Sci..

[42] Haque M., Li Q., Kuzmenko V., Smith A.D., Enoksson P. (2017). Ionic liquid electrolyte for supercapacitor with high temperature compatibility. J. Phys. Conf. Ser..

[43] He X., Yang C.P., Zhang G.L., Shi D.W., Huang Q.A., Xiao H.B., Liu Y., Xiong R. (2016). Supercapacitor of TiO_2_ nanofibers by electrospinning and KOH treatment. Mater. Des..

[44] Li H., Wu D., Zhu X., Yang C., Liu D., Chen X., Song Y., Lu L. (2014). High-performance and renewable supercapacitors based on TiO_2_ nanotube array electrodes treated by an electrochemical doping approach. Electrochim. Acta.

[45] Vidyadharan B., Archana P.S., Ismail J., Yusoff M.M., Jose R. (2015). Improved supercapacitive charge storage in electrospun niobium doped titania nanowires. RSC Adv..

[46] Samet M., Levchenko V., Boiteux G., Seytre G., Kallel A., Serghei A. (2015). Electrode polarization vs. Maxwell-Wagner-Sillars interfacial polarization in dielectric spectra of materials: Characteristic frequencies, and scaling laws. J. Chem. Phys.

[47] Asandulesa M., Kostromin S., Aleksandrov A., Tameev A., Bronnikov S. (2022). The effect of PbS quantum dots on molecular dynamics and conductivity of PTB7:PC71BM bulk heterojunction as revealed by dielectric spectroscopy. Phys. Chem. Chem. Phys..

[48] Pochard I., Vall M., Eriksson J., Farineau C., Cheung O., Frykstrand S., Welch K., Stromme M. (2018). Amine-functionalised mesoporous magnesium carbonate: Dielectric spectroscopy studies of interactions with water and stability. Mater. Chem. Phys..

[49] Asandulesa M., Kostromin S., Tameev A., Aleksandrov A., Bronnikov S. (2021). Molecular dynamics and conductivity of a PTB7:PC71BM photovoltaic polymer blend: A dielectric spectroscopy study. ACS Appl. Polym. Mater..

[50] Larsson O., Said E., Berggren M., Crispin X. (2009). Insulator polarization mechanisms in polyelectrolyte-gated organic field-effect transistors. Adv. Funct. Mater..

