# Supplementary material for: Multi-Functional Materials Based on Cu-Doped TiO2 Ceramic Fibers with Enhanced Pseudocapacitive Performances and Their Dielectric Characteristics
Source: Polymers (Basel). 2022 Nov 4;14(21):4739. doi: 10.3390/polym14214739 (PMC9654394; doi:10.3390/polym14214739)
Supplement: Supplementary file 1 [file polymers-14-04739-s001.zip › polymers-1993095-supplementary.pdf]

Multi-functional materials based on Cu doped TiO_2 ceramic fibers with enhanced pseudocapacitive performances and their dielectric characteristics

Petronela Pascariu*, Mihaela Homocianu, Loredana Vacareanu, Mihai Asandulesa

”Petru Poni” Institute of Macromolecular Chemistry, 41A Grigore Ghica Voda Alley, 700487, Iasi, Romania

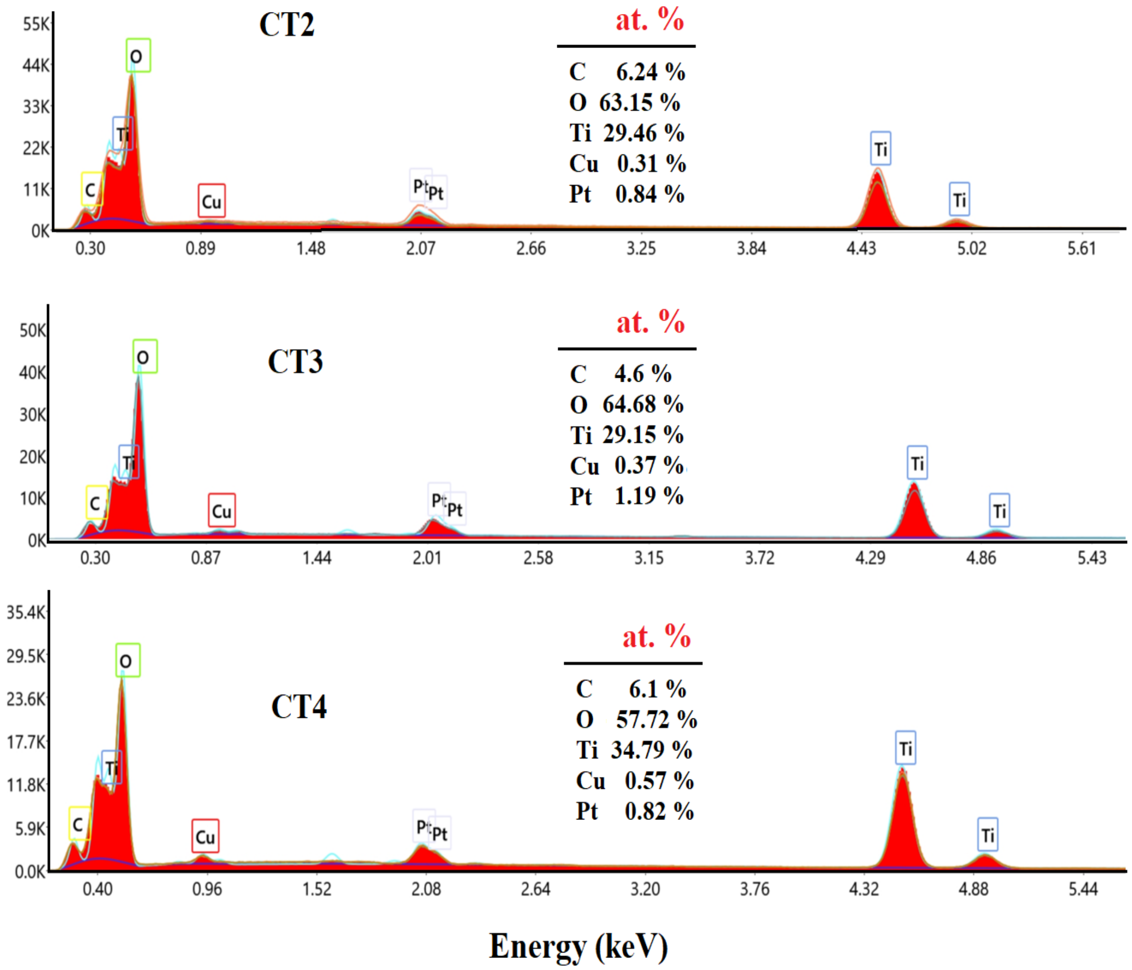

Figure S1. EDS spectra of 0.5% Cu (CT2), 1% Cu (CT3), 2% Cu (CT4) doped TiO_2 composites.

*Corresponding author: dorneanu.petronela@icmpp.ro; pascariu_petronela@yahoo.com (Petronela Pascariu);

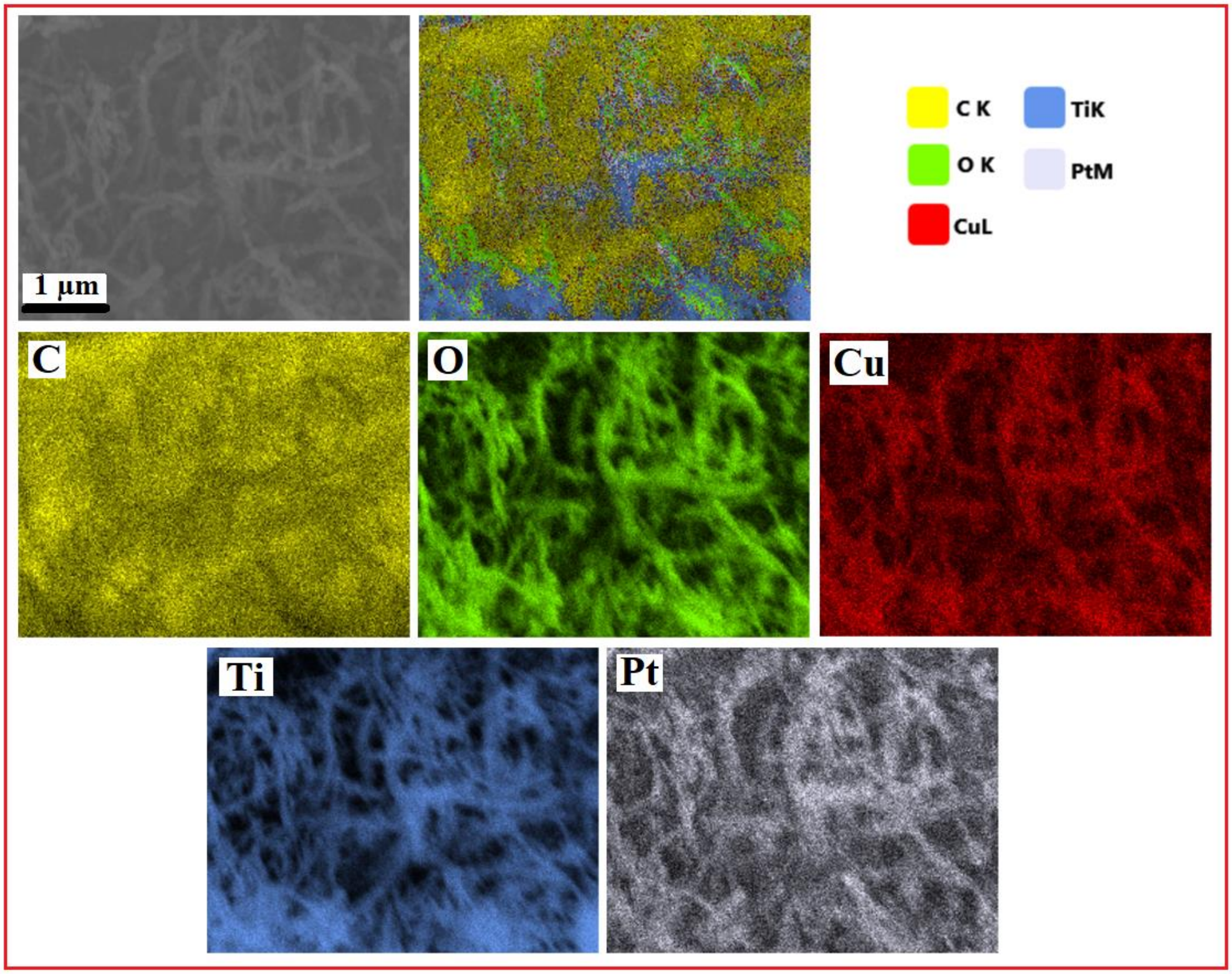

Figure S2. Elemental mapping images of 2% Cu- doped TiO_2 (CT4) composite.

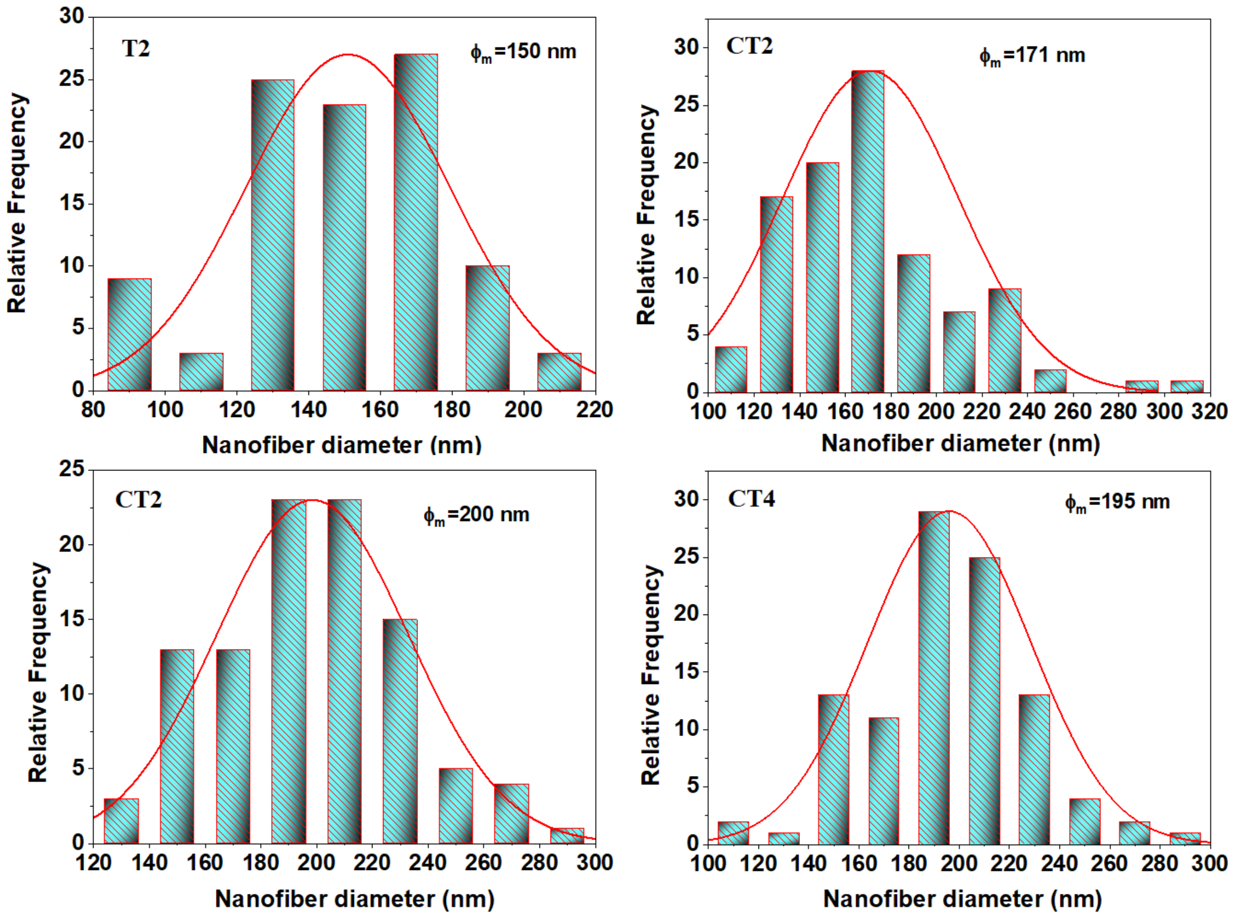

Figure S3. Histogram plots of average size diameter distribution for pure TiO_2 and 0.5% Cu (CT2), 1% Cu (CT3), 2% Cu (CT4) doped TiO_2 composites.
